# Biological behaviors of muscarinic receptors in mesenchymal stem cells derived from human placenta and bone marrow

**DOI:** 10.22038/IJBMS.2019.38582.9151

**Published:** 2020-01

**Authors:** Arash Alizadeh Yegani, Erkan Maytalman, Ilknur Kozanoglu, Menderes Yusuf Terzi, Fazilet Aksu

**Affiliations:** 1 Hatay Mustafa Kemal University, Department of Pharmacology and Toxicology, Hatay, Turkey; 2 Alanya Alaaddin Keykubat University, Department of Medical Pharmacology, Antalya, Turkey; 3 Başkent University, Faculty of Medicine, Adult Bone Marrow Transplant Center, Adana, Turkey; 4 Hatay Mustafa Kemal University, Faculty of Medicine, Department of Medical Biology, Hatay, Turkey; 5 Cukurova University, Faculty of Medicine, Department of Medical Pharmacology, Adana, Turkey

**Keywords:** Atropine, Bone marrow, Cell differentiation, Gene expression, Human placenta, Mesenchymal stemcells, Muscarinic receptors

## Abstract

**Objective(s):**

Cells perform their functional activities by communicating with each other through endogenous substances and receptors. Post-translation, stem cells function properly in new host tissue by carrying specific cell surface receptors. We aimed to characterize muscarinic receptor subtypes in mesenchymal stem cells (MSCs) together with osteogenic and adipogenic differentiation markers.

**Materials and Methods:**

mRNA levels of 5 muscarinic receptor subtypes (*CHRM1 *to* 5*), BMP-6, and *PPARγ* during osteogenic and adipogenic differentiation, under the effect of atropine blockade, were measured in MSCs obtained from human fetal membrane (FM) and bone marrow (BM). Additionally, the effect of atropine on differentiation in the 1st, 2nd, and 3rd passages of MSCs, obtained from human FM and BM, were analyzed by RT-qPCR.

**Results:**

*CHRM1* mRNA levels increased in the FM group, while decreasing in the BM group. We found significant decreases in *CHRM3* and *CHRM5* mRNA levels in FM and BM groups, respectively. Atropine had variable effects based on cell source and receptor type. *BMP-6* mRNA levels in differentiated osteogenic cells increased significantly compared to undifferentiated cells in both FM and BM groups. In MSCs derived from both sources, *PPARγ* mRNA levels in differentiated adipogenic cells increased significantly. Atropine showed no effect on MSCs differentiation.

**Conclusion:**

These results indicate that expressions of muscarinic receptors in MSCs derived from BM and FM can vary and these cells keep the potential of osteogenic and adipogenic differentiation *in vitro*. Besides, atropine had no effect on adipogenic and osteogenic differentiation of MSCs.

## Introduction

Today, studies with mesenchymal stem cells (MSCs) offer novel approaches as a therapeutic tool in clinics with an increasing frequency due to both availability and the use in regenerative medicine (1, 2). MSCs were identified and isolated from bone marrow (BM) in the 1970s for the first time by Friedenstein and his colleagues (1). MSCs are multipotent stem cells that are present in several tissues including umbilical cord blood, adipose tissue, adult muscle, corneal stroma (3), peripheral blood (4), placenta (5), etc. MSCs are self-renewable, easily accessible, and culturally expandable cells, which emphasizes their importance in cell therapy, regenerative medicine, and repairman of tissue (6). MSCs, when exposed to the appropriate stimuli, are differentiated into several mesenchymal lineages such as chondrocytes, osteocytes, skeletal muscle cells, and adipocytes under specific cell culture conditions (7, 8). These specifications of MSCs are regulated by a variety of signaling pathways, for example β-FGF (9), Wnt 3a (10), bone morphogenic proteins (BMPs) (11), Notch (12), epidermal growth factor receptor (EGFR) (13), IL-6 (14), and others such as acetylcholine receptors (AChRs), which have not been explored yet. 

Acetylcholine (ACh), acetyltransferase, acetylcholinesterase, and AChRs constitute the cholinergic system. These molecules are expressed in several non-neural cell types such as embryonic stem cells (ES) (15), hematopoietic stem cells (HSC) (16), neural stem cells (NSC) (17, 18), MSCs (19-21), and skeletal muscle stem cells (SMSC) (22). Thus it is suggested that ACh plays a role in regulating the functions of various stem cells (e.g., NSC, HSC, SMSC) such as proliferation and differentiation (16, 18, 22). 

MSCs have been often investigated regarding expression patterns of several cell surface markers such as adhesion molecules and immunological hematopoietic markers (23). MSCs are positive for both nicotinic (nAChRs) and muscarinic receptors (mAChRs). Muscarinic receptors, consisting of five subtypes, which are cholinergic receptor muscarinic 1 (*CHRM1*) to *CHRM5*, mediate postganglionic parasympathetic cholinergic signal transduction (23). ACh regulates apoptosis (24, 25) but not proliferation of MSCs (19). Importantly, mAChRs play a role in the migration of various cell types (26, 27). However, the function of the cholinergic system in the regulation of MSC migration is not fully known yet. There is also limited number of studies about the expression pattern of mAChRs in MSCs with respect to primary culturing, passaging, and differentiation of these particular cells. Besides, the expression profiles of mAChRs in MSCs derived from different sources are poorly understood. Furthermore, the effect of mAChRs blockade on differentiation and other cellular activities of MSCs, such as viability, proliferation, etc., is in need of detail investigation. In an *in vitro* study, the effect of atropine on muscarinic receptor activation and epithelial-mesenchymal transition was analyzed in the lung epithelial cell line (28). In another study, they stimulated bone marrow-derived regenerated cardiomyocyte (CMG) cells with muscarinic receptors agonist carbachol, and the cells were treated with atropine as muscarinic receptor blocker (29). Such studies characterizing MSCs with respect to mAChRs have a higher priority to investigate which primary source and at which differentiation level the MSCs can be utilized for more successful transplantation in patient-specific clinical therapies. In this manner our aim in this study is to investigate the expression of mAChRs in MSCs obtained from different sources *in vitro* and the effect of atropine, as a mAChR blocker, on MSC differentiation. 

## Materials and Methods

***Cell culture preparations of MSCs from placenta tissue and bone marrow***

Human placentas from healthy donor mothers were obtained from mothers undergoing cesarean section delivery after written and informed consent at the Department of Gynecology and Obstetrics, University of Baskent Hospital Adana, Turkey (n=3). Bone marrow aspirates drawn from human posterior iliac crest were obtained from the Adult Bone Marrow Transplantation Center, Başkent University in Adana, after getting written and informed consents of the donors (n=3). For the present study approval was obtained from the Ethics Committee of Non-Interventional Clinical Research (GOKEAK) of Cukurova University (Turkey).

The amnion was carefully separated from the chorion, and the amnion was immediately washed with phosphate-buffered saline (PBS, Stemcell Technologies, Canada) solution to remove blood and mucus. Then the tissue was minced into small fragments (0.5 –1 cm), which were then treated with 25 ml 0.25% trypsin-EDTA supplemented with 1% penicillin and streptomycin solution (Stemcell Technologies, Canada) and incubated at least for a week in a 37 °C, 5% CO2 humidified incubator (Thermo Scientific, Heracell 150i, Germany). Thereafter non-adherent cells were removed by refreshing the culture medium (MesenCult™ Proliferation Kit (Human), Stemcell Technology, Canada) including fetal bovine serum (FBS, Gibco, UK) for trypsin inactivation. After 70% to 80% confluence was achieved, adherent MSCs were harvested via trypsinization, and the cell suspension was used for the following experiments.

Under sterile conditions, aspirated bone marrow was delivered into sterile conical tubes and then transferred into a 15 ml culture medium (MesenCult™ Proliferation Kit (Human), Stemcell Technology, Canada). After four days, the medium was replenished gently to keep MSCs adhered to the culture dish surface. For further experiments we waited for the cells to reach 80% confluence that is approx. 1x10^6^ cells per T75 flask (Thermo Scientific, USA). 

After 14 days (P0) in culture, adherent MSCs were harvested via trypsinization, and the cell fraction was passaged three times for further experiments. The osteogenic and adipogenic differentiation were observed under the effect of atropine at each passage. The experimental groups of fetal membrane MSCs (FM-MSCs) and bone marrow MSCs (BM-MSCs) were designated as follows: fetal membrane/bone marrow 1^st^, 2^nd^, 3^rd^ passage groups (FM/BM-P1,-P2,-P3), fetal membrane/bone marrow osteogenic/adipogenic differentiation group (FM-O/FM-A, BM-O/BM-A), fetal membrane/bone marrow osteogenic/adipogenic differentiation group treated with atropine (FM-O-ATR/FM-A-ATR, BM-O-ATR/BM-A-ATR).

***Flow cytometry analysis***

Both types of MSCs were characterized with fluorescence-activated cell sorting (BD FACSCanto™ II, Becton Dickinson, USA) analysis to confirm the expression of CD73^+^ and CD105^+^ as well as documenting lack of CD34^-^ and CD45^-^ expression. MSC’s specific surface antigens were analyzed with flow cytometry in the cell cultures at 1^st^, 2^nd^, and 3^rd^ passages. The MSCs were harvested via trypsinization for each passage and resuspended in culture medium. After centrifugation, the cell pellet was washed with PBS and centrifuged at 300 g for 8 min. For further analyses, the cells were resuspended with 200 µl PBS to get a final 5000 cells/μl density. Monoclonal antibodies used for these experiments were diluted as follows: CD105 FITC (1:20), CD34 PE-Cy7 (1:20), CD73-PE (1:10), and CD45-APC (1:10) (Becton Dickinson, USA). Data were analyzed with FACSDiva software (Ver. 6.1.2).

***MTT proliferation assay and effective blocker dose determination***

MTT assay measures the ability of live cells to convert tetrazolium salt into purple formazan which was used for determination of a safe dose of atropine. Since atropine effect on cell viability regarding MSCs has not been evaluated before, we needed to perform MTT assay in our study. The effect of atropine sulfate salt monohydrate (Sigma, USA) on cell viability was tested in the 1^st^ passage. Effective atropine dose determination was measured in standard cell culture. FM-MSCs and BM-MSCs were seeded in 96-well plates (Thermo Scientific, USA, 100 µl, 1x10^5^ cells⁄ml) and treated with atropine at the following concentrations: 0.1, 1, 5, 10, 50, 100, and 500 µM. After incubation with atropine for a total of 7 days, by refreshing the medium every 48 hr, the viability of MSCs cells was assessed. After seven days, 10 µl MTT solution (5 mg/ml, Sigma-Aldrich, Germany), prepared with fresh PBS, was added into each well and incubated for 4 hr at 37 ^°^C. The cell culture medium was replaced, and 100 µl, 99,9% dimethyl sulfoxide (DMSO) (Wak-Chemie Gmbh, Germany) was added into each well. Optical density values were measured with a microplate reader (Bio-Tek Inc., USA) at 570 nm.

***In vitro differentiation studies***

Cultured MSCs were cultured in T25 flasks with a 2x10^4 ^cell/ml concentration. All differentiation experiments took 21 days (three weeks), and the culture medium was replenished twice per week. Additionally, 1 µM atropine was supplemented into the cell differentiation media in order to analyze the blocker effect of atropine on cell differentiation.

Adipogenesis was stimulated by culturing MSCs in MesenCult™ Adipogenic Differentiation Medium (Stemcell Technologies, Canada) for three weeks according to the manufacturer’s instructions. Adipogenic differentiation of FM-MSCs and BM-MSCs was evaluated from passages 2 to 3 with Oil Red O (Sigma, USA) staining, which indicates intracellular lipid accumulation as described before (30). After photographing, Oil Red O elutes were harvested from the cultures by using 60% isopropanol and examined under a microscope.

Osteogenesis was stimulated by culturing MSCs for three weeks using MesenCult™ Osteogenic Stimulatory Kit (Stemcell Technologies, Canada). The culture medium was refreshed every three days. FM-MSCs and BM-MSCs for osteogenic differentiation from passages 2 to 3 were evaluated with Alizarin Red S (Sigma, USA) staining, as described before (30), which is specific to calcium. Based on standard protocol for Alizarin Red S staining, osteogenesis levels were analyzed by detecting calcium deposits in cultures. Three experiments were performed using MSCs obtained from three different donors.

At the end of the culture periods, the cells were harvested from T25 flasks for further RNA isolation, cDNA synthesis, and RT-qPCR experiments.

***Quantitative reverse transcription-polymerase chain reaction (RT-qPCR)***

Total RNA was isolated from independent biological replicates of MSCs (n=3), adipocytes (n=3), and osteocytes (n=3) based on manufacturer’s instructions (High Pure RNA Isolation Kit, Roche, Germany). The purity and concentration of RNA were determined by measuring the absorbance at A260/A280 nm in a Nanodrop spectrophotometer (Thermo Scientific, USA).

In the second part of cDNA synthesis, 500 ng total RNA in a 200 µl reaction volume was used following a cDNA synthesis kit protocol (Ipsogen RT Kit CE, Qiagen, USA). The conditions for cDNA synthesis were as follows: 10 min at 25 ^°^C, 60 min at 50 ^°^C , 5 min at 85 ^°^C , and 4 min at 4 ^°^C .

In the final part, quantitative PCR was performed by using SYBR green PCR kit (Applied Biosystems, Foster City, CA, USA and performed on a Rotor-Gene Q (Qiagen, Germany) as follows: step 1: at 95 ^°^C,10 min, step 2: 40 cycles of at 95 ^°^C, 15 sec and at 60 ^°^C, 30 sec. The primers that were purchased from Qiagen were used to amplify the following genes (Table 1): *CHRM1*, *CHRM2*, *CHRM3*, *CHRM4*, *CHRM5*, *BMP-6*, *PPAR-gama*, and β-actin. The expression levels of each target gene (normalized to β-actin) were calculated with the 2^(–∆∆CT)^ relative quantification method.

***Statistical analysis***

Regarding MTT assays, the difference between mean cell viability percentages among groups was calculated with one way ANOVA and *post hoc* Bonferroni tests. MTT experiments were repeated nine times. The MTT data were expressed as percentage (%)±SEM.

To analyze gene expression data (RT-qPCR), the online service of Qiagen (The Gene Globe Data Analysis Center) was used. We used the “Delta Delta Ct (∆∆Ct)” method (internal control β-actin gene for normalization), and the expression levels of target genes were expressed as “fold-change” with the formula 2^(–∆∆CT)^. All gene expression experiments were repeated three times per gene. Groups were compared and analyzed with Student’s t-test. The gene expression data were expressed as mean±SEM.

For statistical significance *P*-value was set as *P* ≤ 0.05. All statistical analyses were performed using Graph Pad Prism ver.7 software.

## Results

***Flow cytometry***

**Figure 1:**
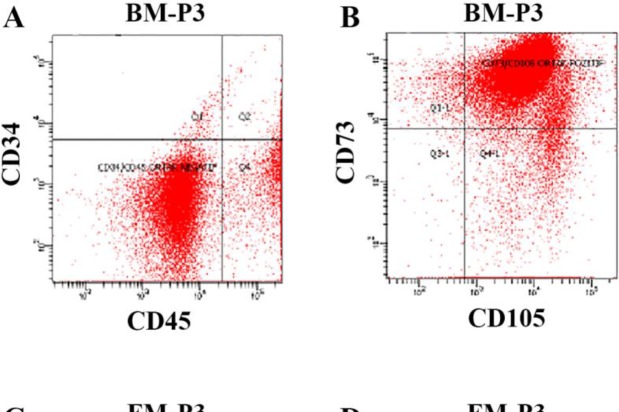
Representative flow cytometry count charts. Expression percentages of hematopoietic (CD34, CD45) and cell surface markers (CD73, CD105) of (A), (B) BM-MSCs and (C), (D) FM-MSCs at P3 (MSC: Mesenchymal stem cell, BM: Bone marrow, FM: Fetal membrane, P3: Cell line passages 3).

The cell lines derived from FM and BM tissues were analyzed at three passages regarding cell surface markers of MSCs with flow cytometry. The MSCs at no passage numbers expressed hematopoietic cell surface markers CD34 and CD45 while they did express MSCs-specific adhesion markers CD73 and CD105 (Figure 1, Table 2).

***Effect of atropine on cell viability***

**Figure 2:**
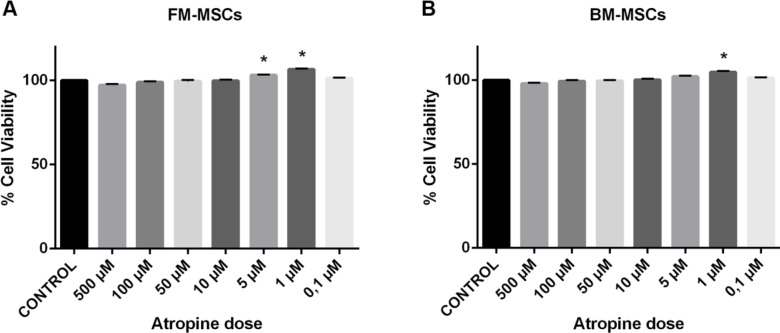
Effect of atropine on the cell viability of (A) FM-MSCs and (B) BM-MSCs. Cell viability of untreated control was set to 100%, and the treated groups were calculated based on the control. FM-MSCs: Fetal membrane mesenchymal stem cells, BM-MSCs: Bone marrow mesenchymal stem cells. The data were expressed as percentage (%)±SEM. (* *P*<0.05, n=9)

The MTT tests demonstrated the effect of atropine at the 5 tested doses on the cell viability of BM-MSCs and FM-MSCs. The results of the test groups were expressed as percentages of the control, which represents 100% cell viability. All results obtained with MTT tests showed that 1 μM atropine in both groups and 5 μM atropine in FM-MSCs showed a significant increase in the viability of MSCs (*P<*0.05, Figures 2A and 2B). For further gene expression analyses to test blocker effect of atropine, 1 μM dose was selected.

***mRNA levels of muscarinic receptors in FM-MSCs and BM-MSCs differentiation after atropine treatment***

**Figure 3:**
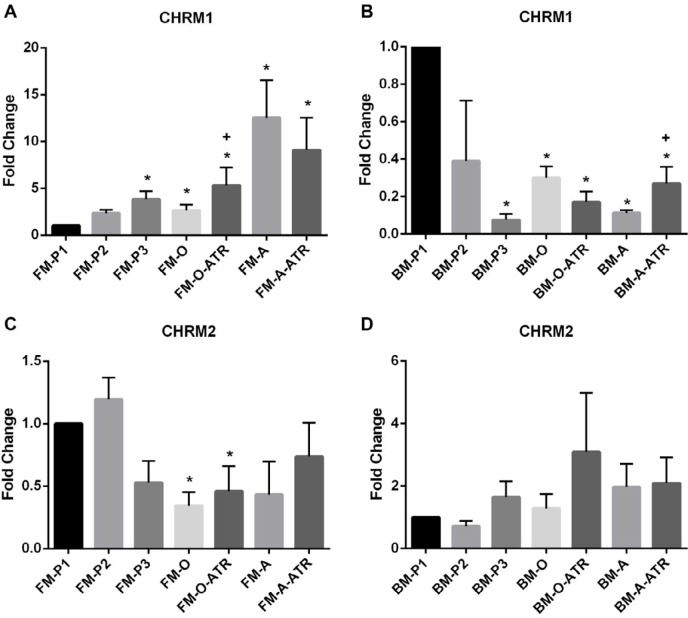
*CHRM1* and *CHRM2* mRNA levels in (A,C) FM-MSCs and (B,D) BM-MSCs. FM: Fetal membrane; BM: Bone marrow, P1, P2, P3: cell culture passages 1, 2, 3; -O: Osteogenic differentiation, -A: Adipogenic differentiation, -ATR: Atropine treatment during cell differentiation. The data were expressed as mean±SEM. * *P*<0.05, n=3 (vs FM-P1 or BM-P1). + *P*<0.05 (For *CHRM1* expression; FM-O-ATR vs FM-O and BM-A-ATR vs BM-A)

The expression of *CHRM1* in FM-MSCs groups showed that *CHRM1* mRNA levels in all groups except FM-P2 were significantly higher compared to the FM-P1 as the control (*P<*0.05, Figure 3A). Also the expression of *CHRM1* in FM-O-ATR was significantly higher than FM-O (*P<*0.05, Figure 3A).

In the BM-MSCs groups, mRNA levels of *CHRM1* in all groups except BM-P2 were significantly lower compared to the BM-P1 control (Figure 3B, *P<*0.05). Besides, the expression of *CHRM1* in BM-A-ATR was significantly higher than BM-A (*P<*0.05, Figure 3B).

The expression of *CHRM2* in the BM-MSCs group did not show any significant difference (Figure 3D), but it significantly decreased in FM-O and FM-O-ATR groups compared to FM-P1 (*P<*0.05, Figure 3C).

**Figure 4:**
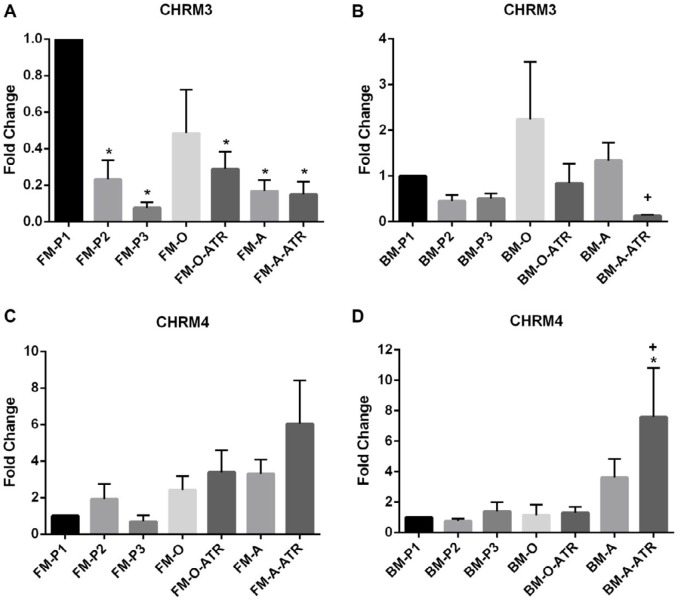
*CHRM3* and *CHRM4* mRNA levels in (A,C) FM-MSCs and (B,D) BM-MSCs. FM: Fetal membrane; BM: Bone marrow, P1, P2, P3: cell culture passages 1, 2, 3; -O: Osteogenic differentiation, -A: Adipogenic differentiation, -ATR: Atropine treatment during cell differentiation. The data were expressed as mean±SEM. * *P*<0.05, n=3 (vs FM-P1 or BM-P1). + *P*<0.05 (For *CHRM3* and *CHRM4* expressions; BM-A-ATR vs BM-A)

*CHRM3* expression in all FM-MSCs groups except FM-O significantly decreased. Regarding BM-MSCs groups, there was no significant change. However, *CHRM3* mRNA level in BM-A-ATR was significantly lower than BM-A (*P<*0.05, Figure 4B).

Regarding FM-MSCs groups, there was no significant difference between *CHRM4* expression levels. As to BM-MSCs groups, a significant increase was found in just the BM-A-ATR group compared to the control (*P<*0.05, Figure 4D). *CHRM4* expression in BM-A-ATR was also significantly higher than BM-A (*P<*0.05, Figure 4D).

**Figure 5:**
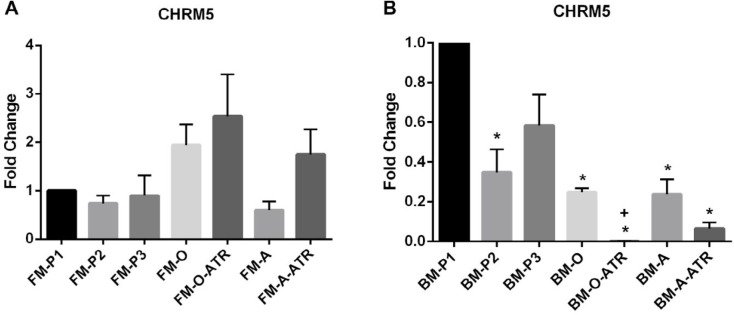
*CHRM5* mRNA level in (A) FM-MSCs and (B) BM-MSCs. FM: Fetal membrane; BM: Bone marrow, P1, P2, P3: cell culture passages 1, 2, 3; -O: Osteogenic differentiation, -A: Adipogenic differentiation, -ATR: Atropine treatment during cell differentiation. The data were expressed as mean±SEM. * *P*<0.05, n=3 (vs FM-P1 or BM-P1). + *P*<0.05 (BM-O-ATR vs BM-O)

The expression of *CHRM5* in all FM-MSCs groups showed no significant difference (Figure 5A). Conversely, regarding BM-MSCs, expression of *CHRM5* decreased significantly in all groups except BM-P3 compared to FM-P1 (*P<*0.05, Figure 5B). Besides, with respect to differences among six experimental groups, expression of *CHRM5* in BM-O-ATR was significantly lower than BM-O (*P<*0.05) (Figure 5B).

***mRNA levels of BMP-6 and PPAR-gama in FM-MSCs and BM-MSCs differentiation after atropine treatment***

*BMP-6* and *PPAR-gama* expressions in osteogenic and adipogenic differentiations were tested by using MSCs derived from FM and BM with the RT-qPCR method. In order to assess the effect of atropine on differentiation of MSCs bone morphogenetic protein-6 (*BMP-6*) as osteogenic marker and peroxisome proliferator-activated receptor-gamma (*PPAR-gama*) as adipogenic differentiation marker were investigated. 

**Figure 6:**
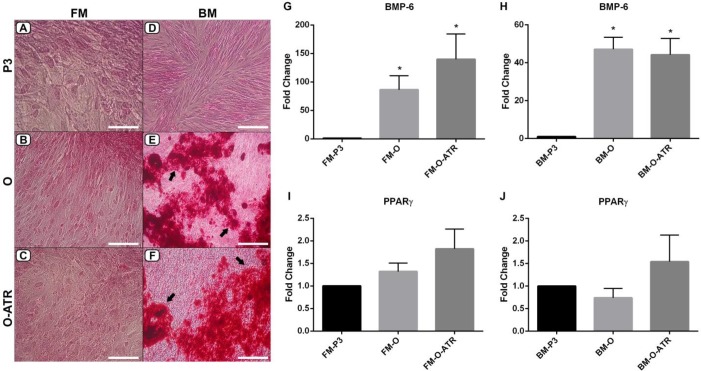
Alizarin red S staining indicating the calcium deposition and osteogenic differentiation in (A,B,C) FM-MSCs and (D,E,F) BM-MSCs. (A) FM-P3, (B) FM-O, (C) FM-O-ATR, (D) BM-P3 (undifferentiated), (E) BM-O, (F) BM-O-ATR. Magnification/Scale bars: A, x400, 50 µm; B,C,F, x200, 100 µm; D,E, x100, 200 µm.* BMP-6* and *PPAR-gamma* mRNA level in (G,I) FM-MSCs and (H,J) BM-MSCs. FM: Fetal membrane; BM: Bone marrow, P3: cell culture passage 3; -O: Osteogenic differentiation, -ATR: Atropine treatment. *P<0.05, n=3 (vs FM-P3 or BM-P3)

Regarding *BMP-6* and *PPAR-gama* expressions in osteogenic differentiation, the mRNA levels of *BMP-6* in the groups of FM-O and FM-O-ATR were found to be significantly higher compared to the FM-P3 control (*P<*0.05, Figure 6G). Besides, a significantly higher *BMP-6* expression was found in BM-O and BM-O-ATR groups compared to the BM-P3 control (*P<*0.05, Figure 6H). No significant differences in *PPAR-gama* expressions were observed in either FM-MSCs and BM-MSCs groups (Figures 6I and 6J). There was also no significant atropine effect in the expression levels of *BMP-6* during the osteogenic differentiation.

**Figure 7:**
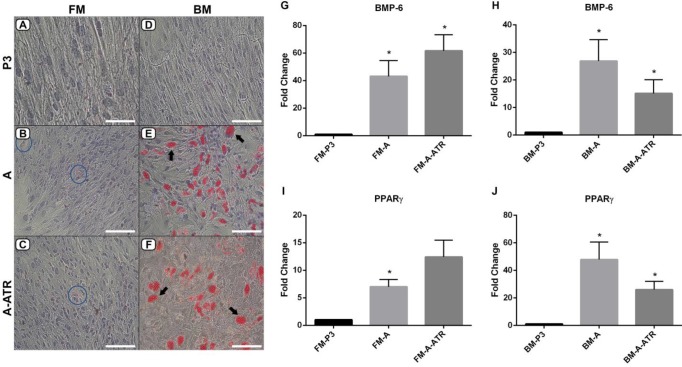
Oil red O staining indicating the lipid droplets and adipogenic differentiation in (A,B,C) FM-MSCs and (D,E,F) BM-MSCs. (A) FM-P3, (B) FM-A, (C) FM-A-ATR, (D) BM-P3, (E) BM-A, (F) BM-A-ATR. Magnification/Scale bars: A, x400, 50 µm; B,C,D,E,F, x200, 100 µm. *BMP-6* and *PPAR-gamma* mRNA level in (G,I) FM-MSCs and (H,J) BM-MSCs. FM: Fetal membrane; BM: Bone marrow, P3: cell culture passage 3; -A: Adipogenic differentiation, -ATR: Atropine treatment during cell differentiation. * *P*<0.05, n=3 (vs FM-P3 or BM-P3)

Regarding *BMP-6* and *PPAR-gama* expressions in adipogenic differentiation, we specifically analyzed the mRNA expression of *PPAR-gama*, as an adipogenic marker, to determine the blocker effect of atropine on the differentiation of MSCs. Consistent with the *PPAR-gama* expression profile, except FM-A-ATR group, *BMP-6* expression increased in FM-MSCs and BM-MSCs groups compared to the FM-P3 and BM-P3 control groups, respectively (*P<*0.05, Figures 7G and 7H). The expression of *PPAR-gama* in FM-A increased significantly compared to the FM-P3 control (*P<*0.05, Figure 7I). Besides, the *PPAR-gama* expression levels also showed a significant increase in the BM-MSCs groups (*P<*0.05, Figure 7J).

***Morphological analysis of osteogenic differentiation***

We investigated the morphology of the MSCs after osteogenic differentiation in BM-MSCs and FM-MSCs groups and analyzed the effect of atropine on osteogenic differentiation in these groups. For this purpose, we performed Alizarin Red S staining to examine the effect of atropine on osteogenic differentiation.

In contrast to *BMP-6* mRNA level results in FM-MSCs groups (Figure 6G), the morphology of FM-O (Figure 6B) and FM-O-ATR (Figure 6C) showed no mineralized nodule formation (calcium deposition) compared to the FM-P3 control (Figure 6A). The stained nodules in BM-O and BM-O-ATR groups (black arrows, Figures 6E and 6F) were also analyzed, and it was observed that calcium deposition in these groups was more notable compared to the BM-P3 control in parallel with the *BMP-6* expression pattern (Figures 6D and 6H). In addition, we observed no significant effect of atropine on either gene expression or morphological alteration during osteogenic differentiation process.

***Morphological analysis of adipogenic differentiation***

The differentiation of adipocytes was evaluated under a microscope with Oil red O Staining, which labels lipid droplets. BM-A and BM-A-ATR (arrows, Figures 7E and 7F) groups exhibited more lipid accumulation than the BM-P3 control (Figure 7D). However, the stained lipid droplets of adipocytes in FM-MSCs were less spread out than the ones in BM-MSCs. In contrast, the FM-MSCs groups demonstrated a dispersed pattern for cytoplasmic fat droplets and a notable morphological change in the same time frame. Adipocyte transformation of the cells in FM-A and FM-A-ATR (circles, Figures 7B and 7C) groups was notable unlike the FM-P3 control (Figure 7A). In terms of adipogenic differentiation, there was an increase in *BMP-6* expression levels in both FM-MSCs and BM-MSCs groups compared to the controls (Figures 7G and 7H). Besides, *PPAR-gamma* expression levels showed a significant increase, except FM-A-ATR, in BM-MSCs and FM-MSCs groups, which was supported by morphological analysis (Figures 7A-F and 7I-J). Despite this, the stained lipid droplets in FM-MSCs were less than the ones in BM-MSCs (Figure 7A-F). Moreover, the effect of atropine on adipogenic differentiation in FM-MSCs and BM-MSCs groups was not significant with respect to both gene expression and morphological perspectives (Figure 7).

## Discussion

Nowadays, stem cells obtained from different sources are used for regenerative purposes in preclinical and clinical studies by converting them into different cell types to treat many diseases and to eliminate tissue-organ damage. In the present study, fetal membrane- (FM) and bone marrow (BM)-derived MSCs in the 1^st^, 2^nd^, and 3^rd^ passages were cultured to investigate the osteogenic and adipogenic differentiations, as well as the effect of atropine during this process, by analyzing the mRNA levels of *CHRM1*, *CHRM2*, *CHRM3*, *CHRM4*, *CHRM5* muscarinic receptor subtypes, *BMP-6*, and *PPAR-gama* with RT-qPCR method. 

In a previous study, doses between 0.1 and 10 μM of atropine were used to analyze the effect of muscarinic receptor activation on the epithelial-mesenchymal transition in the lung epithelial cell line (28). In another study, atropine at a dose of 1 μM was used to test the antagonist effect in MSCs obtained from mouse BM (29). In these studies mentioned above, no preliminary study was performed to determine the safe dose of atropine. Therefore, first of all we determined the effect of atropine on the viability of FM-MSCs and BM-MSCs with MTT assay. After atropine treatment, we observed no cytotoxic effect at any concentrations. On the contrary, we found a significant increase in cell viability at 1 μM and 5 μM doses in FM-MSCs and 1 μM dose in BM-MSCs. So, we decided to use 1 μM of atropine since it resulted in the most efficient viability rate. Atropine was detected for the first time in this study as exerting a propagative effect on cell viability at a dose of 1 μM in MSCs obtained from different sources.

In an *in vitro* study, the function of the cholinergic system was demonstrated in a murine embryonic stem cell line, which implies the overt role of the cholinergic system during early developmental stages (15). In another study, the presence of the CHRM2-receptor was demonstrated on human MSCs (19). In addition, it was previously shown that the mRNA levels of *CHRM1* and *CHRM2* muscarinic receptors in cardiomyocytes cells were markedly inhibited by atropine and AFDX116 (29). The present study is the first to show that the MSCs obtained from different sources act disparately regarding the expressions of muscarinic receptors and morphological features due to the passage number and differentiation processes even if the cells were derived from the same source. We obtained that, the mRNA levels of *CHRM1* muscarinic receptors varied among different sources that MSCs were derived from. Furthermore, according to our gene expression data, *CHRM1* muscarinic receptors seemed to have a functional role, especially in the adipogenic differentiation of FM-MSCs cells. We detected that *CHRM1* receptors fluctuated in different directions as increase or decrease in both FM and BM sources. While significant increases were noted in *CHRM1* expression in FM-MSCs, BM-MSCs had decreased expression levels. Atropine treatment caused a significant increase compared to FM-O and BM-A groups regarding *CHRM1* expression. Our results suggest that increased mRNA levels of *CHRM1* receptor imply its functional role in the cells of FM-MSCs groups. Besides, mRNA levels of *CHRM2* receptors in FM-MSCs showed significant decreases in osteogenic groups, but no significant change was found in any of the BM-MSCs groups. In terms of *CHRM2*, there were significant differences in both FM-O and FM-O-ATR groups. But there was no significant atropine effect on the expression levels. We showed with these results that *CHRM1* and *CHRM2* had different expression patterns in the cells derived from various sources.

There are significant decreases in *CHRM3* mRNA levels during the passaging and differentiation stages of FM-MSCs while there was no alteration in BM-MSCs, which may indicate that *CHRM3* receptor may have diverse functions based on origin of source during differentiation process. *CHRM4* mRNA levels did not show any significant change in the FM and BM groups compared to the controls. As for atropine effect, whereas it suppressed the expression of CHRM3 in adipogenic BM cells, it showed a contrary effect regarding CHRM4 receptor expression in the same cell types. But the increase in *CHRM4* expression, despite atropine blockade in BM-A-ATR group of cells, indicates the importance of the *CHRM4* muscarinic receptor during adipogenic differentiation process. No significant difference was found in *CHRM5* mRNA levels in FM groups. But in BM groups, significant decreases in *CHRM5* expression were observed compared to the controls. These results show that muscarinic receptors are differentially expressed in the cells depending on the cell types that are originated from different sources. Besides, the fluctuations in the levels of different muscarinic receptor subtypes in both cell types can be an intrinsic regulatory mechanism to compensate and maintain the physiological levels of the receptors within the cell.

In the second part of the study, morphological and molecular changes of the MSCs were evaluated during cellular differentiation by using *BMP-6* as osteogenesis and *PPAR-gama* as adipogenesis marker. A study conducted by Li *et al.* (31) investigated the expression of *PPAR-gama* to determine the effect of isoproterenol on the adipogenic differentiation of MSCs. They found that during adipogenesis of BM-derived MSCs, β-adrenergic receptors up-regulated and were implicated in the adipogenic differentiation of MSCs and isoproterenol inhibited this process in a time and dose-dependent manner (31). In another *in vitro* study, it was found that MSCs had higher osteogenic differentiation capacity than normal cells, and upregulation of *BMP-2* and *BMP-6* augmented alkaline phosphatase levels (32). In our study, mRNA levels of *BMP-6* and *PPAR-gama* were also determined to analyze the effect of atropine, as the receptor blocker, on osteogenic and adipogenic differentiation. In osteogenic differentiation, the mRNA level of *BMP-6*, which is an important protein for the osteogenic differentiation, was significantly higher compared to the undifferentiated control cells. The increased *BMP-6 *levels in BM-MSCs were also approved by morphological analyses, but it was not the case for FM-MSCs. The difference between morphologies and *BMP-6* levels can be caused by some secondary pathways that affect the presence of BMP-6 protein in cells, which needs to be further analyzed in this stage. With respect to *PPAR-gama* expression, as an adipogenic marker, in FM-O and BM-O groups, no significant difference was detected compared to the undifferentiated control as expected. Besides, there was also no effect of atropine on the expression levels of *BMP-6* and *PPAR-gama* as well as in morphologies in both MSCs groups. In terms of adipogenic differentiation, the *PPAR-gama* expression levels showed a significant increase in BM-MSCs and FM-MSCs groups, except FM-A-ATR. Morphologically analysis of BM-MSCs is in line with the increased *PPAR-gama* levels, but scarcely stained lipid droplets in FM-MSCs were not as distinct as the ones in BM-MSCs. The inconsistency between the increased *PPAR-gama* mRNA levels in FM-A and the weak staining pattern of lipid droplets might arise from some underlying pathways that should be investigated. As similar to the osteogenic differentiation, atropine exerted no solid effects regarding either gene expression levels or morphological differentiation in both MSCs groups.

**Table 1:** Gene symbols and gene-specific primers (Qiagen, Germany)

Gene symbols	Primer codes	Product size (bp)	Annealing (°C)	Cycle		Reference sequences
CHRM1	PPH02686F	107 bp	60	40x	NM_000738	
CHRM2	PPH02690C	92 bp	60	40x	NM_000739	
CHRM3	PPH02721A	108 bp	60	40x	NM_000740	
CHRM4	PPH02691A	123 bp	60	40x	NM_000741	
CHRM5	PPH02687A	191 bp	60	40x	NM_012125	
BMP-6	PPH00542F	80 bp	60	40x	NM_001718	
PPAR-γ	PPH02291G	93 bp	60	40x	NM_005037	
β-Actin	PPH00073G	174 bp	60	40x	NM_001101	

**Table 2:** Flowcytometry data indicating average expression patterns of hematopoietic (CD34, CD45) and cell surface markers (CD73, CD105) of MSCs (MSC: Mesenchymal stem cell, BM: Bone marrow, FM: Fetal membrane, P1, 2, and 3: Cell line Passages 1, 2, and 3)

MSC source	Immunophenotypic markers (%)			
	CD 34	CD45	CD73	CD105
BM-P1	3,2	1,9	96,1	96,2
BM-P2	0,7	1,6	95,8	99,6
BM-P3	0,4	0,5	99,3	99,1
FM-P1	0,1	1,9	96,7	92,6
FM-P2	0,1	0,6	99,3	96,4
FM-P3	0,2	0,3	98,9	99,9

## Conclusion

The FM is an appropriate MSC source for obtaining a large number of cells, which are easier to get and more numerous than the cells obtained from BM and other organs/tissues. FM is also a more suitable stem cell source in terms of ethical rules because it is considered as waste after birth. According to our results, muscarinic receptor genes revealed various gene expression patterns with respect to the source of MSCs. We observed consistency between *BMP-6* and *PPAR-gama* mRNA levels and the morphological differentiation of BM-MSCs. In contrast, although FM-derived cells also underwent osteogenic and adipogenic differentiation at the gene level, they were not phenotypically well-differentiated as BM-derived cells. At last but not least, whereas we found variable effects of atropine on some of the muscarinic receptor gene expressions in both MSC sources, it did not interfere with the osteogenic and adipogenic differentiation processes of MSCs.
